# Impact of a switch to fingolimod versus staying on glatiramer acetate or beta interferons on patient- and physician-reported outcomes in relapsing multiple sclerosis: *post hoc* analyses of the EPOC trial

**DOI:** 10.1186/s12883-014-0220-1

**Published:** 2014-11-26

**Authors:** Jonathan Calkwood, Bruce Cree, Heidi Crayton, Daniel Kantor, Brian Steingo, Luigi Barbato, Ron Hashmonay, Neetu Agashivala, Kevin McCague, Nadia Tenenbaum, Keith Edwards

**Affiliations:** Minneapolis Clinic of Neurology, Golden Valley, MN USA; University of California San Francisco, San Francisco, CA USA; MS Center of Greater Washington, Vienna, VA USA; Neurologique, Ponte Vedra, FL USA; Fort Lauderdale MS Center, Pompano Beach, FL USA; Novartis Pharmaceuticals Corporation, East Hanover, NJ USA; MS Center of Northeastern New York, Latham, NY USA

**Keywords:** 36-item Short-Form Health Survey (SF-36), Beck Depression Inventory-II (BDI-II), Clinical Global Impressions of Improvement (CGI-I), Fatigue Severity Scale (FSS), Fingolimod, FTY720, Multiple sclerosis, Treatment Satisfaction Questionnaire for Medication (TSQM)

## Abstract

**Background:**

The Evaluate Patient OutComes (EPOC) study assessed physician- and patient-reported outcomes in individuals with relapsing multiple sclerosis who switched directly from injectable disease-modifying therapy (iDMT; glatiramer acetate, intramuscular or subcutaneous interferon beta-1a, or interferon beta-1b) to once-daily, oral fingolimod. *Post hoc* analyses evaluated the impact of a switch to fingolimod versus staying on each of the four individual iDMTs.

**Methods:**

Overall, 1053 patients were randomized 3:1 to switch to fingolimod or remain on iDMT. The primary endpoint was the change in Treatment Satisfaction Questionnaire for Medication (TSQM) Global Satisfaction score. Secondary endpoints included changes in scores for TSQM Effectiveness, Side Effects and Convenience subscales, Beck Depression Inventory-II (BDI-II), Fatigue Severity Scale (FSS), Patient-Reported Outcome Indices for Multiple Sclerosis (PRIMUS) Activities, 36-item Short-Form Health Survey (SF-36) Mental Component Summary (MCS) and Physical Component Summary (PCS) and mean investigator-reported Clinical Global Impressions of Improvement (CGI-I). All outcomes were evaluated after 6 months of treatment.

**Results:**

Changes in TSQM Global Satisfaction scores were superior after a switch to fingolimod when compared with scores in patients remaining on any of the iDMTs (all *p* <0.001). Likewise, all TSQM subscale scores improved following a switch to fingolimod (all *p* <0.001), except when compared with glatiramer acetate for the TSQM Side Effects subscale (*p* = 0.111). FSS scores were found to be superior for fingolimod versus remaining on subcutaneous interferon beta-1a and interferon beta-1b, BDI-II scores were significantly improved for fingolimod except for the comparison with intramuscular interferon beta-1a, and SF-36 scores were superior with fingolimod compared with remaining on interferon beta-1b (MCS and PCS; *p* = 0.030 and *p* = 0.022, respectively) and subcutaneous interferon beta-1a (PCS only; *p* = 0.024). Mean CGI-I scores were superior with fingolimod when compared with continuing treatment with any of the iDMTs (all *p* <0.001).

**Conclusions:**

After 6 months, a switch to fingolimod showed superiority compared with remaining on each iDMT for a range of patient- and physician-reported outcomes, including global satisfaction with treatment.

**Trial registration:**

ClinicalTrials.gov NCT01216072.

## Background

Fingolimod is a disease-modifying therapy (DMT) that is administered as an oral tablet [1]; it is the first once-daily, oral immunotherapy for the treatment of relapsing multiple sclerosis (MS) approved by the US Food and Drug Administration. Relapsing MS is a disease characterized by phases of neurological deficits followed by stable periods, eventually resulting in accumulated neurological deterioration that increases disability [2,3] and reduces quality of life (QOL) [4]. Fingolimod exerts its therapeutic effects via modulation of sphingosine 1-phosphate receptors on T-lymphocytes, which results in the selective and reversible retention of naïve and central memory T-lymphocytes within lymph nodes, preventing their circulation to other tissues, including the central nervous system.

Prior to the approval of fingolimod, treatment of MS commonly used injectable DMTs (iDMTs) such as beta interferons (IFNs) or glatiramer acetate (GA). However, patients face various issues with use of self-administered injections of these therapies, such as anxiety over the use of needles, tolerability problems and injection-site side effects relating to long-term use [5,6]. In addition, iDMTs have shown limited efficacy in some patients [7,8].

The phase 3 TRANSFORMS and FREEDOMS trials have demonstrated fingolimod to be superior to placebo and intramuscular (IM) IFN beta-1a in reducing relapse rate and in terms of magnetic resonance imaging (MRI) measures [9,10]. Evidence that fingolimod significantly improves health-related QOL and patient-reported outcomes (PROs) in comparison with placebo has also been reported [11]. In order to determine the impact of fingolimod versus active comparators on health-related QOL, the open-label Evaluate Patient OutComes (EPOC; NCT01216072) study was conducted. EPOC was the first trial comparing a switch to fingolimod versus remaining on any of four iDMTs (either GA or one of three IFN betas) on a range of physician-related outcomes and PROs [12]. EPOC showed that, after switching to fingolimod therapy for 6 months, patients had significant improvements in most self-reported outcomes when compared with those who continued to receive an iDMT, including satisfaction with treatment, fatigue severity, depression severity, physical function and mental health [13]. In addition, by including a broader patient population than was included in the registration trials, and by allowing treatments to be switched with no washout period, the EPOC study has provided information regarding a treatment switch in a real-world scenario.

These *post hoc* analyses examined the effects of a therapy switch to fingolimod on the different outcome measures assessed in the EPOC trial and compared them with remaining on each of the four individual iDMTs. The rationale for conducting the current study was that the primary analysis was restricted to comparison of fingolimod with iDMTs as a single group, whereas the *post hoc* analyses presented here focused on the comparison of a switch to fingolimod from each individual iDMT versus remaining on each individual iDMT. This study therefore aimed to determine whether there were specific iDMTs for which patients would benefit from a switch of therapy.

## Methods

### Study design

EPOC was a 6-month, randomized, open-label, multicenter, phase 4 study conducted in the USA and Canada. Patients were randomized 3:1 to switch to fingolimod (FTY720; Gilenya®, Novartis Pharma AG, Basel, Switzerland) 0.5 mg or remain on/switch to an iDMT for 6 months with no intervening washout period. The primary analysis evaluated two groups, namely fingolimod versus any iDMT. Patients randomized to the iDMT group either remained on the same therapy or, following consultation with a physician, were switched immediately to another approved iDMT. The four iDMTs were subcutaneous (SC) IFN beta-1b (Extavia®, Novartis Pharma AG, Basel, Switzerland, or Betaseron®, Bayer AG, Leverkusen, Germany) 0.25 mg every other day, IM IFN beta-1a (Avonex®, Biogen Idec, Cambridge, MA, USA) 30 μg once weekly, SC IFN beta-1a (Rebif®, Merck Serono, Darmstadt, Germany, and Pfizer Inc., New York City, NY, USA) 22 or 44 μg three times weekly, or SC GA (Copaxone®, Teva Pharmaceutical Industries Ltd, Petah Tikva, Israel) 20 mg once daily. The protocol and informed consent form were reviewed and approved by an institutional review board (Quorum Review) at each study center, and every patient provided written informed consent.

### Patient inclusion criteria

Men and women aged 18–65 years with relapsing forms of MS, as defined by the 2005 revised McDonald criteria [14], and an Expanded Disability Status Scale (EDSS) score of 0–5.5 were eligible to participate in the study. Patients were required to have received a single iDMT (except natalizumab) continuously for at least 6 months prior to study initiation and to be candidates for therapy change. For patients from the USA, the treating physician determined whether the patient was a suitable candidate for therapy change. In the case of Canadian patients, only those with relapsing–remitting MS and who had an inadequate response to, or were unable to tolerate, one or more therapies for MS were eligible. Patients were required to have been naïve to fingolimod treatment.

### Patient exclusion criteria

Patients were excluded from the study for any of the following reasons: chronic immune system disease other than MS; immunodeficiency; malignancy other than localized basal cell carcinoma within the past 5 years; a history of cardiac arrest, myocardial infarction, ischemic heart disease or coronary spasm within the past 6 months; Mobitz type II second-degree heart block, third-degree atrioventricular block or an increased corrected QT (QTc) interval (>470 ms); having undergone a bone marrow transplant; a history of alcohol abuse within the past 5 years. Further exclusion criteria at the time of screening were: macular edema; active systemic infection; a negative test for Varicella zoster immunoglobulin G antibodies; positive tests for hepatitis B, hepatitis C or human immunodeficiency virus; tuberculosis; uncontrolled diabetes; uncontrolled or poorly controlled hypertension or asthma; cardiac failure; severe respiratory disease or pulmonary fibrosis; chronic liver or biliary disease. Patients were also excluded if they had been treated with the following medications: immunosuppressants, immunoglobulins or monoclonal antibodies within the 6 months before screening; any live or live attenuated vaccines during the month before screening; cladribine, cyclophosphamide or mitoxantrone at any time; class Ia or class III antiarrhythmic drugs at the time of screening.

### Endpoints

For the primary and secondary *post hoc* study endpoints, individual comparisons were conducted for all outcome measures for switching to fingolimod compared with remaining on any of the four individual iDMTs.

#### Primary endpoint

The primary *post hoc* study endpoint was the least-squares mean (LSM) change in Treatment Satisfaction Questionnaire for Medication (TSQM) Global Satisfaction score from baseline to 6 months [15]. This was measured using the Global Satisfaction subscale score on the TSQM v1.4, where higher scores indicate greater satisfaction.

#### Secondary endpoints

LSM changes from baseline to 6 months were calculated for the TSQM Effectiveness, Side Effects and Convenience subscale scores, again using TSQM v1.4. In the event that more than one item was missing from a subscale score of the TSQM, the subscale was considered invalid.

The 10-item Fatigue Severity Scale (FSS) was used to assess fatigue severity and its effects on daily living, with higher scores indicating greater fatigue severity [16]. The Beck Depression Inventory-II (BDI-II), which contains 21 multiple-choice questions, was used to evaluate changes in patient-reported depression during the trial; higher scores indicate greater severity of depression [17]. The Patient-Reported Outcome Indices for Multiple Sclerosis (PRIMUS) Activities, a 15-item assessment [18], was used to evaluate changes in activities of daily living, with higher scores indicating greater activity limitation. For all of these instruments, the result was specified as ‘missing’ if more than 20% of the total number of scores were absent.

Health-related QOL was evaluated using the 36-item Short-Form Health Survey (SF-36) v2, which is a self-administered questionnaire measuring eight domains of health [19]. These include physical functioning, role limitations due to physical health, bodily pain, general health perceptions, vitality, social functioning, role limitations due to emotional problems and general mental health. Higher scores indicate a better QOL. The SF-36 allows the calculation of two summary scale scores: the Mental Component Summary (MCS) and the Physical Component Summary (PCS). For any of the eight domains, if more than half of the questions were not answered, the score for that domain was stated as ‘missing’; furthermore, the MCS and PCS were also stated as ‘missing’ if any of the eight scale scores were absent.

A further secondary endpoint, the physician-rated Clinical Global Impressions of Improvement (CGI-I), provided a global evaluation of clinical change over 6 months. Using this scale, the physician scores a patient’s level of improvement between 1 (very much improved since the initiation of treatment) and 7 (very much worse since the initiation of treatment) [20].

### Statistical analyses

For the primary endpoint, enrollment of 1000 patients (750 fingolimod; 250 iDMT) would provide 90% power to detect a significant difference between fingolimod and each iDMT group in the change from baseline, assuming an effect size of 0.25, a significance level of 5% and a 10% rate of unevaluable patients.

*Post hoc* analyses were conducted using SAS software v9.2 (SAS, Cary, NC, USA). For the analysis of individual iDMTs, the primary variable was assessed using analysis of covariance, with baseline TSQM Global Satisfaction score as a covariate and treatment group as a main effect, as was done for the analysis of the pooled iDMTs [13]. The differences in LSM change from baseline to 6 months between the treatment groups are reported, with the exception of CGI-I where the differences between the mean scores after 6 months of treatment are reported. Missing data were imputed using the last observation carried forward.

## Results

### Patient baseline characteristics

Study enrollment was completed in October 2011. Of 1053 patients randomized to treatment, 790 were randomized to fingolimod 0.5 mg and 263 were randomized to an iDMT. Of the patients switched to fingolimod, 262 switched from GA, 205 switched from IM IFN beta-1a, 196 switched from SC IFN beta-1a and 125 switched from IFN beta-1b. For patients who remained on an iDMT, 74 remained on GA, 48 remained on IM IFN beta-1a, 58 remained on SC IFN beta-1a and 39 remained on IFN beta-1b. Patients who switched from one iDMT to another or who had been taking a therapy other than one of the four study iDMTs prior to randomization were not included in the analysis.

The eight study arms had similar baseline demographic and disease characteristics (Table 1). The majority of patients in each group were women (69.9–84.6%) and Caucasian (75.0–86.5%), and the mean age ranged from 44.4 to 47.5 years (absolute range 18–65 years). In the year before screening, the mean number of MS relapses was between 0.48 and 0.88 across all arms. The mean duration of MS symptoms was 11.1–13.1 years from the point of first symptom appearance and the mean EDSS score was 2.3–2.5 across all groups.

**Table 1:** **Patient demographics and baseline characteristics**

	**GA to fingolimod**	**Remaining on GA**	**IM IFN beta-1a to fingolimod**	**Remaining on IM IFN beta-1a**	**SC IFN beta-1a to fingolimod**	**Remaining on SC IFN beta-1a**	**IFN beta-1b to fingolimod**	**Remaining on IFN beta-1b**
	**(n = 262)**	**(n = 74)**	**(n = 205)**	**(n = 48)**	**(n = 196)**	**(n = 58)**	**(n = 125)**	**(n = 39)**
**Mean age (SD), years**	46.3 (9.14)	44.4 (9.97)	46.6 (9.90)	45.1 (10.48)	45.0 (10.39)	45.9 (10.25)	46.3 (10.20)	47.5 (8.97)
**Women, n (%)**	208 (79.4)	61 (82.4)	160 (78.0)	38 (79.2)	137 (69.9)	44 (75.9)	94 (75.2)	33 (84.6)
**Race, n (%)**								
Caucasian	222 (84.7)	64 (86.5)	165 (80.5)	36 (75.0)	160 (81.6)	44 (75.9)	94 (75.2)	31 (79.5)
Black	24 (9.2)	9 (12.2)	35 (17.1)	10 (20.8)	29 (14.8)	14 (24.1)	24 (19.2)	6 (15.4)
Asian	2 (0.8)	0 (0.0)	0 (0.0)	0 (0.0)	0 (0.0)	0 (0.0)	1 (0.8)	0 (0.0)
Native American	2 (0.8)	0 (0.0)	0 (0.0)	1 (2.1)	2 (1.0)	0 (0.0)	0 (0.0)	0 (0.0)
Other	12 (4.6)	1 (1.4)	5 (2.4)	1 (2.1)	5 (2.6)	0 (0.0)	6 (4.8)	2 (5.1)
**Mean duration of MS symptoms (SD), years**	13.1 (8.91)	12.2 (9.36)	12.0 (7.9)	11.5 (7.87)	11.1 (7.89)	11.4 (8.04)	12.2 (8.64)	12.3 (6.78)
**Mean number of MS relapses (SD)**								
Previous year	0.75 (5.53)	0.84 (3.12)	0.74 (5.54)	0.48 (1.66)	0.76 (6.30)	0.88 (3.64)	0.74 (3.90)	0.62 (1.84)
Previous 2 years	1.42 (9.60)	1.43 (5.81)	1.20 (7.78)	0.88 (2.72)	1.29 (8.54)	1.45 (5.05)	1.34 (5.50)	1.05 (2.83)
**Mean EDSS score (SD)**	2.5 (1.33)	2.4 (1.35)	2.5 (1.26)	2.4 (1.27)	2.4 (1.35)	2.3 (1.38)	2.4 (1.37)	2.5 (1.39)

EDSS, Expanded Disability Status Scale; GA, glatiramer acetate; IFN, interferon; IM, intramuscular; MS, multiple sclerosis; SC, subcutaneous; SD, standard deviation.

### Primary endpoint by treatment

**Figure 1:**
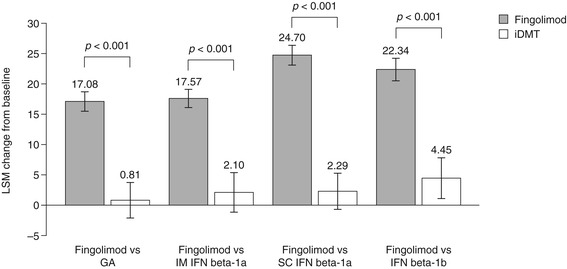
**Change in Treatment Satisfaction Questionnaire for Medication Global Satisfaction scores.** The figure shows the LSM change from baseline to 6 months ± standard error. GA, glatiramer acetate; IFN, interferon; iDMT, injectable disease-modifying therapy; IM, intramuscular; LSM, least-squares mean; SC, subcutaneous.

LSM changes (± standard error) in TSQM Global Satisfaction scores from baseline to 6 months were significantly superior following a switch to fingolimod compared with remaining on GA (17.08 ± 1.56 versus 0.81 ± 2.89, respectively), IM IFN beta-1a (17.57 ± 1.51 versus 2.10 ± 3.22, respectively), SC IFN beta-1a (24.70 ± 1.62 versus 2.29 ± 2.96, respectively) or IFN beta-1b (22.34 ± 1.85 versus 4.45 ± 3.29, respectively); all *p* <0.001. Results are shown in Figure 1.

### Secondary endpoints by treatment

#### Treatment satisfaction questionnaire for medication Effectiveness, Side Effects and Convenience subscales

LSM changes in scores for TSQM Effectiveness (12.16 ± 1.49 for fingolimod versus 0.62 ± 2.76 for GA; 13.31 ± 1.48 for fingolimod versus 1.37 ± 3.13 for IM IFN beta-1a; 15.07 ± 1.65 for fingolimod versus 1.62 ± 3.02 for SC IFN beta-1a; 17.59 ± 2.06 for fingolimod versus 0.68 ± 3.62 for IFN beta-1b), Side Effects (30.62 ± 1.56 for fingolimod versus 6.56 ± 3.27 for IM IFN beta-1a; 27.83 ± 1.43 for fingolimod versus −0.42 ± 2.61 for SC IFN beta-1a; 21.50 ± 1.88 for fingolimod versus −1.24 ± 3.30 for IFN beta-1b) and Convenience (38.01 ± 0.80 for fingolimod versus 3.11 ± 1.49 for GA; 43.83 ± 0.94 for fingolimod versus 5.70 ± 1.97 for IM IFN beta-1a; 42.36 ± 1.02 for fingolimod versus 1.66 ± 1.87 for SC IFN beta-1a; 41.57 ± 1.15 for fingolimod versus 1.31 ± 2.04 for IFN beta-1b) were superior at 6 months following a switch to fingolimod compared with remaining on any of the four iDMTs (all *p* <0.001), with the exception of TSQM Side Effects score for fingolimod versus GA (9.25 ± 1.41 versus 4.47 ± 2.64, respectively; *p* = 0.111). Results are shown in Table 2.

**Table 2:** **Change from baseline to 6 months for TSQM Effectiveness, Side Effects and Convenience subscale scores**

	**GA to fingolimod**	**Remaining on GA**	**IM IFN beta-1a to fingolimod**	**Remaining on IM IFN beta-1a**	**SC IFN beta-1a to fingolimod**	**Remaining on SC IFN beta-1a**	**IFN beta-1b to fingolimod**	**Remaining on IFN beta-1b**
	**(n = 262)**	**(n = 74)**	**(n = 205)**	**(n = 48)**	**(n = 196)**	**(n = 58)**	**(n = 125)**	**(n = 39)**
**Effectiveness**	12.16 ± 1.49	0.62 ± 2.76	13.31 ± 1.48	1.37 ± 3.13	15.07 ± 1.65	1.62 ± 3.02	17.59 ± 2.06	0.68 ± 3.62
**Side effects**	*9.25 ± 1.41*	*4.47 ± 2.64*	30.62 ± 1.56	6.56 ± 3.27	27.83 ± 1.43	−0.42 ± 2.61	21.50 ± 1.88	−1.24 ± 3.30
**Convenience**	38.01 ± 0.80	3.11 ± 1.49	43.83 ± 0.94	5.70 ± 1.97	42.36 ± 1.02	1.66 ± 1.87	41.57 ± 1.15	1.31 ± 2.04

Values are displayed as least-squares means ± standard errors. *p* <0.001 for all comparisons; italics = non-significant result.

GA, glatiramer acetate; IFN, interferon; IM, intramuscular; SC, subcutaneous; TSQM, Treatment Satisfaction Questionnaire for Medication.

#### Beck depression inventory-II

**Figure 2:**
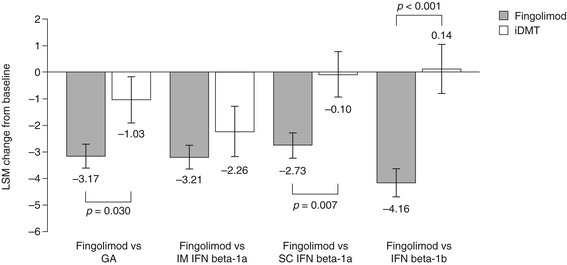
**Change in Beck Depression Inventory-II scores.** The figure shows the LSM change from baseline to 6 months ± standard error. GA, glatiramer acetate; iDMT, injectable disease-modifying therapy; IFN, interferon; IM, intramuscular; LSM, least-squares mean; SC, subcutaneous.

LSM changes in BDI-II scores from baseline to 6 months were significantly superior following a switch to fingolimod compared with remaining on GA (−3.17 ± 0.46 versus −1.03 ± 0.86, respectively; *p* = 0.030), SC IFN beta-1a (−2.73 ± 0.47 versus −0.10 ± 0.86, respectively; *p* = 0.007) or IFN beta-1b (−4.16 ± 0.52 versus 0.14 ± 0.93, respectively; *p* <0.001). No significant differences in LSM changes were observed between switching to fingolimod and remaining on IM IFN beta-1a (−3.21 ± 0.44 versus −2.26 ± 0.93, respectively; *p* = 0.358). Results are shown in Figure 2.

#### Fatigue severity scale

**Figure 3:**
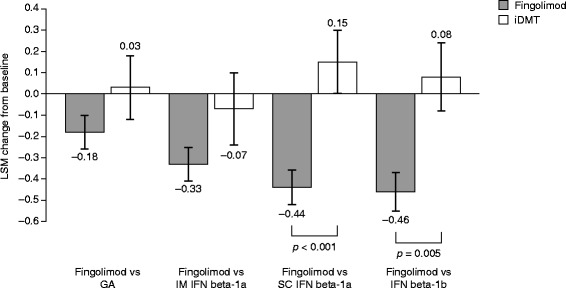
**Change in Fatigue Severity Scale scores.** The figure shows the LSM change from baseline to 6 months ± standard error. GA, glatiramer acetate; iDMT, injectable disease-modifying therapy; IFN, interferon; IM, intramuscular; LSM, least-squares mean; SC, subcutaneous.

LSM changes in FSS scores from baseline to 6 months were significantly superior following a switch to fingolimod compared with remaining on SC IFN beta-1a (−0.44 ± 0.08 versus 0.15 ± 0.15, respectively; *p* <0.001) and IFN beta-1b (−0.46 ± 0.09 versus 0.08 ± 0.16, respectively; *p* = 0.005). No significant differences in LSM changes were observed between switching to fingolimod and remaining on GA (−0.18 ± 0.08 versus 0.03 ± 0.15, respectively; *p* = 0.218) or IM IFN beta-1a (−0.33 ± 0.08 versus −0.07 ± 0.17, respectively; *p* = 0.179). Results are shown in Figure 3.

#### Patient-reported outcome indices for multiple sclerosis activities

LSM changes in PRIMUS Activities scores from baseline to 6 months were not significantly different between patients switched to fingolimod compared with those remaining on GA (−0.25 ± 0.31 versus −0.40 ± 0.57, respectively; *p* = 0.818), IM IFN beta-1a (−0.51 ± 0.28 versus −0.90 ± 0.59, respectively; *p* = 0.554), SC IFN beta-1a (−0.77 ± 0.29 versus 0.06 ± 0.54, respectively; *p* = 0.182) or IFN beta-1b (−0.93 ± 0.30 versus 0.03 ± 0.52, respectively, *p* = 0.111).

#### 36-item short-form health survey

##### Mental component summary

**Figure 4:**
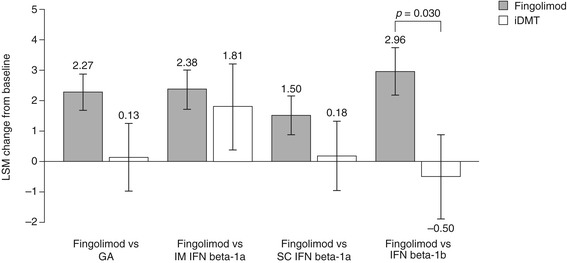
**Change in 36-item Short-Form Health Survey Mental Component Summary scores.** The figure shows the LSM change from baseline to 6 months ± standard error. GA, glatiramer acetate; iDMT, injectable disease-modifying therapy; IFN, interferon; IM, intramuscular; LSM, least-squares mean; SC, subcutaneous.

LSM changes in SF-36 MCS scores from baseline to 6 months were significantly superior following a switch to fingolimod compared with remaining on IFN beta-1b (2.96 ± 0.76 versus −0.50 ± 1.39, respectively, *p* = 0.030). No significant differences in LSM changes were observed between switching to fingolimod and remaining on GA (2.27 ± 0.59 versus 0.13 ± 1.11, respectively; *p* = 0.089), IM IFN beta-1a (2.38 ± 0.67 versus 1.81 ± 1.41, respectively; *p* = 0.719) or SC IFN beta-1a (1.50 ± 0.63 versus 0.18 ± 1.14, respectively; *p* = 0.315). Results are shown in Figure 4.

##### Physical component summary

**Figure 5:**
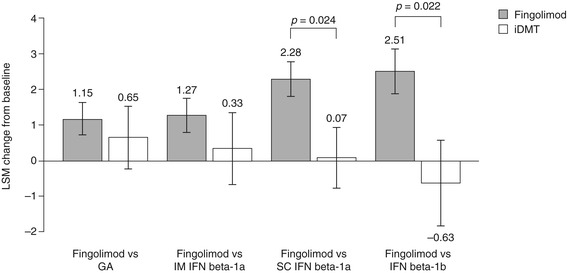
**Change in 36-item Short-Form Health Survey Physical Component Summary scores.** The figure shows the LSM change from baseline to 6 months ± standard error. GA, glatiramer acetate; iDMT, injectable disease-modifying therapy; IFN, interferon; IM, intramuscular; LSM, least-squares mean; SC, subcutaneous.

LSM changes in SF-36 PCS scores from baseline to 6 months were significantly superior following a switch to fingolimod compared with remaining on SC IFN beta-1a (2.28 ± 0.47 versus 0.07 ± 0.85, respectively; *p* = 0.024) or IFN beta-1b (2.51 ± 0.65 versus −0.63 ± 1.19, respectively, *p* = 0.022). No significant differences in LSM changes were observed between switching to fingolimod and remaining on GA (1.15 ± 0.46 versus 0.65 ± 0.87, respectively; *p* = 0.606) or IM IFN beta-1a (1.27 ± 0.48 versus 0.33 ± 1.01, respectively; *p* = 0.400). Results are shown in Figure 5.

#### Clinical global impressions of improvement

**Figure 6:**
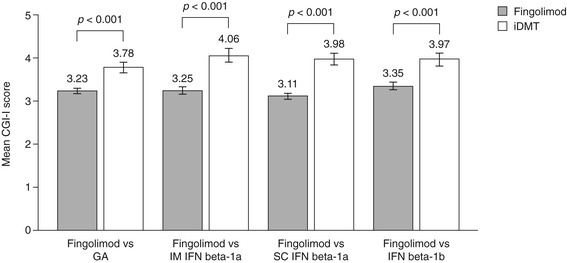
**Clinical Global Impressions of Improvement scores.** The figure shows the mean score for clinician impressions of overall improvement after 6 months of treatment. CGI-I, Clinical Global Impressions of Improvement; GA, glatiramer acetate; iDMT, injectable disease-modifying therapy; IFN, interferon; IM, intramuscular; SC, subcutaneous.

Physicians perceived significantly greater clinical improvement following a switch to fingolimod compared with remaining on GA (CGI-I score at 6 months, 3.23 ± 0.07 versus 3.78 ± 0.13, respectively), IM IFN beta-1a (3.25 ± 0.08 versus 4.06 ± 0.16, respectively), SC IFN beta-1a (3.11 ± 0.07 versus 3.98 ± 0.13, respectively) or IFN beta-1b (3.35 ± 0.09 versus 3.97 ± 0.15, respectively); all *p* <0.001. Results are shown in Figure 6.

## Discussion

These *post hoc* analyses provide the first evidence of a benefit of a switch to fingolimod from an individual iDMT, compared with remaining on each individual iDMT, using a broad spectrum of patient satisfaction and QOL measures, and methodology that is reflective of the real-world use of fingolimod. Perceived improvements in effectiveness and convenience, along with impact on fatigue, depression and mental and physical wellbeing, balanced against perceived tolerability and side effects were evaluated as likely determinants of treatment satisfaction in this study.

The main findings from the *post hoc* analyses support the results of the primary EPOC study, namely that a switch from GA or IFN beta to fingolimod is associated with significantly better scores for the primary endpoint of overall satisfaction with treatment. Here, benefits from a switch of therapy to fingolimod were apparent relative to each of the four iDMTs evaluated, rather than being driven by improvement over only a single therapy or a subset of therapies. The largest change in TSQM Global Satisfaction score was observed as a result of a switch to fingolimod from SC IFN beta-1a.

Patients also reported greater satisfaction following a switch to fingolimod versus remaining on iDMTs in terms of effectiveness, side effects and convenience. TSQM Effectiveness scores were superior for fingolimod compared with all four iDMTs, with the largest change in score seen following a switch from IFN beta-1b. TSQM Side Effects scores were significantly improved following a switch to fingolimod compared with remaining on each of the iDMTs, except for GA. These findings are in line with previous research showing that fingolimod is an effective and generally well-tolerated therapy [9,10,21]. Change in TSQM Convenience scores was significantly greater for fingolimod versus all four iDMTs, with the greatest improvements seen in patients undergoing a switch to fingolimod from IM IFN beta-1a or SC IFN beta-1a. Again, this result is consistent with earlier research showing a higher degree of patient satisfaction and QOL associated with oral versus injectable therapies, as well as adherence issues relating to repeated use of injectable therapies [22-25].

Fatigue outcomes were significantly improved in patients who underwent a switch to fingolimod from SC IFN beta-1a and IFN beta-1b. Fatigue is an extremely common problem for patients with MS, with substantial numbers reporting it to be a moderate-to-severe problem [26-29]. The variable effects on FSS scores shown here are interesting, with superior scores reported for a switch to fingolimod compared with remaining on either of the SC IFN beta therapies, but not for a switch from GA or IM IFN beta-1a. This suggests that individual DMTs impact fatigue differently; this may be due to varying effects on the components of fatigue (e.g. mental versus physical). Alternatively, other factors such as variation in therapy adherence resulting from more or less favorable dosing schedules may explain the pattern of these results [30].

Depressive symptoms significantly improved following a switch to fingolimod compared with remaining on each of the iDMTs, except for IM IFN beta-1a. Several authors report links between depression and fatigue [31-34], with some finding particularly strong associations between depression and the mental component of fatigue [34]. The prevalence of depression is high in patients with MS, and the frequency of major depression exceeds that reported in individuals without MS and in those with other illnesses [35]. Some studies in MS also suggest that increasing disability is associated with worsening BDI-II scores [36], although the root cause of depression in MS is not known with certainty. Better control of depression observed in patients switching to fingolimod (with the exception of the comparison with IM IFN beta-1a, which also appeared to improve BDI-II scores in contrast to findings of previous studies [36]) may therefore result from factors such as better control of disease progression or reduction in fatigue, which may in turn contribute to the improved satisfaction with treatment.

PRIMUS Activities scores showed no significant change as a result of therapy switch to fingolimod from any of the four iDMTs. This may suggest comparable levels of impact of fingolimod and the four iDMTs on ability to conduct daily activities, but it is also possible that the 6-month duration of the EPOC study was not sufficiently long to detect differences between fingolimod and each of the iDMTs. This is an important consideration in light of the significant improvement in the PRIMUS Activities score reported in the 12-month TRANSFORMS phase 3 trial for fingolimod versus IM IFN beta-1a [9].

Changes in mental and physical functioning were significantly greater as a result of switching from IFN beta-1b to fingolimod, and change in PCS score was also superior as a result of a switch to fingolimod from SC IFN beta-1a. The changes in SF-36 MCS and PCS scores as a result of switching to fingolimod support the concept that fingolimod is capable of enhancing mental, emotional, social and/or physical wellbeing in certain subsets of patients receiving iDMTs.

From the physician perspective, a switch to fingolimod was associated with better CGI-I scores irrespective of prior iDMT. Overall, these results indicate that a switch to fingolimod would significantly enhance important aspects of treatment satisfaction and QOL for patients who are receiving these iDMTs. The physician-reported CGI-I results are also reflective of the PROs; this congruence between results is important, as the CGI-I mirrors the content of the PROs used in this study more closely than many traditional physician-determined outcome measures. The latter often focus on biomedical factors (e.g. MRI outcomes) or are weighted towards one particular factor (e.g. mobility in the case of the EDSS score) that may not be fully reflective of how patients feel about the effect of MS on their lives. While these traditional measures are undoubtedly important, a scale such as the CGI-I helps to align physician assessments with those of patient self-assessments, thus helping to facilitate optimal treatment decisions.

One limitation of this study is that these data are unable to offer guidance on a switch in patients who are satisfied with their particular iDMT treatment. It would be of interest to determine whether fingolimod treatment has a similarly significant impact on QOL in patients receiving iDMT, for whom MS is responsive to treatment, and who are tolerant of their current therapy. A second limitation is that the original study was not powered for individual treatment group comparisons; however, most of the comparisons were significant between treatment groups, and provide the first evaluations in these regards between fingolimod and active comparators (GA or IFN beta) as opposed to placebo. With the effect size of 0.25 assumed in the protocol, the power to detect differences in the TSQM Global Satisfaction score between fingolimod and each of the active comparators given the sample sizes seen in the study would range from 27% for IFN beta-1b to 47% for GA. With the samples sizes seen for the subgroups, and assuming 90% power, a range of effect sizes from 0.43 to 0.61 could be detected in the TSQM Global Satisfaction score. Thirdly, as the study utilized an open-label design, lack of concealment had the potential to increase estimates of treatment effects [37]; while the open-label study design provides insight into the outcomes that might be expected in real-world clinical practice, and a control arm was included for objective comparison, findings should be interpreted with caution given the inherent potential for bias in any open-label study. In particular, it should be noted that patients were seeking a therapy switch. Despite this, a switch to fingolimod significantly enhanced patient satisfaction and outcomes scores across a wide range of measures. This suggests that the beneficial effect of a switch is likely a factor of improved efficacy, tolerability and/or superior disease-modifying properties of the therapy rather than purely a result of the psychological impact of switching. It is also possible that factors impacting QOL that are not captured by the outcome measures used in this study may have contributed to the improvements reported by both patients and physicians. Finally, results should also be interpreted with a degree of caution owing to the lack of adjustment for multiplicity; however, in many instances, particularly with TSQM and CGI-I, *p* values are less than 0.001, so these results would still be statistically significant following any method of adjustment for multiplicity.

For future studies, examination of the impact of changing to fingolimod from other DMTs on physician-reported outcomes and PROs would also be of interest. Analyses comparing the effect on relapse rates and MRI outcomes for patients remaining on natalizumab versus a switch to fingolimod in a similar patient population to the EPOC study have recently been completed [38,39], but as yet no data are available for PROs. To help address potential issues from lack of concealment, a study offering the chance to switch from one class of iDMT to another (e.g. GA to IFN beta) may be of interest (a small number of patients not included in these analyses underwent this switch, but sample numbers were too small to conduct meaningful analyses). This would increase confidence that the results were driven by considerations other than simply remaining on a therapy that was poorly tolerated or ineffectual. Subgroup analyses may also be of value in identifying specific patient groups who are eligible for therapy change and who potentially stand to gain the most benefit from a switch to fingolimod for the measures described in this study. Subgroup analyses of the phase 3 FREEDOMS study comparing fingolimod and placebo have identified that most patients, from a patient population with a broad range of clinical characteristics, showed reduced relapse rates and disability progression when treated with fingolimod [40]. These results provide a good rationale for assessing treatment satisfaction and other PROs in subgroups treated with fingolimod versus iDMTs.

## Conclusions

It appears that patients with MS who are candidates for a change in therapy from GA or IFN beta may demonstrate significant improvements in a range of aspects of QOL and PROs by switching to fingolimod therapy. This could therefore have implications for patient preference and physician decisions in the MS population described in this study.

## Abbreviations

BDI-II: Beck Depression Inventory-II
CGI-I: Clinical Global Impression of Improvement
DMT: Disease-modifying therapy
EDSS: Expanded Disability Status Scale
EPOC: Evaluate Patient OutComes
FSS: Fatigue Severity Scale
GA: Glatiramer acetate
iDMT: Injectable disease-modifying therapy
IFN: Interferon
IM: Intramuscular
LSM: Least-squares mean
MCS: Mental Component Summary
MRI: Magnetic resonance imaging
MS: Multiple sclerosis
PCS: Physical Component Summary
PRIMUS: Patient-reported outcome indices for multiple sclerosis
PRO: Patient-reported outcome
QOL: Quality of life
SC: Subcutaneous
SF-36: 36-item Short-Form Health Survey
TSQM: Treatment Satisfaction Questionnaire for Medication
